# Account of the Phænomena Produced by a Large Quantity of Nitric Oxide of Quicksilver, Swallowed by Mistake; and of the Means Employed to Counteract Its Deleterious Influence

**Published:** 1816-03

**Authors:** James Kennedy

**Affiliations:** Dunning, Perthshire


### Journal and Review.

VOL. I.] MARCH, 1816. [NO. 3.

### PART I.

### ORIGINAL COMMUNICATIONS.

*Account of the Phænomena produced by a large Quantity of Nitric Oxide of Quicksilver, swallowed by Mistake; and of the Means employed to counteract its deleterious Influence.* By JAMES KENNEDY, M. D. Dunning, Perthshire.

> Ecce subit virus tacitum, carpitque medullas
> Ignis edax, calidâque incendit viscera tabe.

POISONS, taken into the stomach of animals, induce a train of very alarming phænomena. Their morbific powers, in general, are developed with peculiar vehemency; their influence rapid and afflictive, often fatal. They, at all times, require a prompt administration of the proper antidotes and remedies. Such alone are competent to undertake the treatment, who cultivate medicine as a science, and study the conditions of health and disease as a branch of philosophy.

Mineral poisons act with a dreadful and peculiar energy. Excruciating anguish commonly marks the progress of their operation on the principle of life. For the most part corrosive and stimulant, they produce high constitutional excitement, and inflammation, terminating in disorganization, of the parts with which they come in contact. The knowledge of controlling their destructive operation, and of repairing their ravages on the living structure and functions, can only be improved by the accumulated labours and experience of different observers in the important science of toxicology. As contributing to such an object, I propose to offer a sketch of the symptoms produced by the introduction of a dose of nitric oxide of quicksilver into the stomach of a young man, previously debilitated by disease; and the treatment by which he was fortunately rescued from destruction.

Case. My assistance was requested, in April 1815, by A. B. a farmer's servant, aged 21, unmarried, and of temperate habits. He had been seized, on the preceding day, with frequent and painful vomiting. During the summer of 1814, this young man had suffered from the variolous infection. The fever shortly assumed the typhoid character: the eruption was dense and confluent; the recovery protracted. Greatly enfeebled by the disease, my patient declined active employment, and spent the ensuing autumn and winter with his friends. In the spring of 1815, he returned with renovated zeal and vigour to his rural occupations. Profuse perspiration, induced by an inordinate exertion, was, at this time, suddenly repelled by exposure to a low atmospheric temperature, and large ingestion of cold water. Smart *synocha* was the immediate consequence of this imprudent conduct. I visited the young man in the beginning of March: by the usual means he was soon restored to convalescence; when an accession of fever was again produced by rash exposure to cold and damp. His malady now presented the characteristic marks of *synochus*, and kept him in a state of doubtful recovery during nearly three weeks. Great emaciation and weakness remained: but a sanguine disposition, aided by tonic medicine, nutritious diet, and the genial influence of a mild season, gradually raised to pristine vigour his enervated form; and his countenance resumed the hues of health. Such was the state in which I left him on Saturday evening, the 23d of April, a few hours previously to the occurrence of that unfortunate incident, the circumstances of which it is now my object to communicate.

Late in the evening of the following day, Sunday, I was surprised by a visit from the young man's father. He informed me, that, during the afternoon, his son had been seized with vomiting, attended by exquisite pain in the stomach, and recurring at short intervals. No evacuation had taken place from the rectum. To my repeated inquiries, whether he had taken any other than his accustomed food, or been tampering with some popular remedy, or attempted improper exertion in the earlier part of the day, I received an answer invariably in the negative.

Informed that the ejected matter had a bitter taste and was of a greenish yellow colour, I suspected that biliary derangement, induced by some unknown cause, had excited irritation in the stomach, to which the vomiting and other morbid phænomena might be traced. Such being my views of the case, the suppression of the inordinate action of the gastric organ suggested itself as the primary indication; whereby it might be enabled to retain such remedies as would effectually dislodge the irritating matter. To fulfil this intention, two wine-glassfuls of tepid decoction of barley, rendered grateful by a sweetened solution of gum Arabic, were directed to be drunk every half hour, with a small dose of a mixture of ammoniated tincture of opium and tincture of hyosciamus; and a pill of pure opium to be administered immediately. The irritation of the stomach allayed, five grains of submuriate of mercury were to be given in a pill, and followed, at intervals, by small doses of castor oil, until two ounces of the latter should be consumed. I also directed the abdominal regions to be fomented with flannels wrung out of hot water, and impregnated with laudanum and an aromatic essential oil.

Considering the case to be merely incipient *cholera*, and occupied by another which I deemed to be of a more urgent nature, I contented myself with requesting the old man to see my directions duly fulfilled, and to acquaint me if the situation of his son should become such as to require my personal attendance.

Early on Monday morning, the mother came to inform me that her son had derived no benefit from the medicines; that he now was tormented with frequent alvine evacuations, which excoriated the parts; and was apparently dying. Astonished by this intelligence, I renewed with importunity my former queries; and the unhappy woman unfolded, with grief and horror, the source from which the malady of the young man had evidently arisen.

Among other medicines, he had taken with advantage, during his previous illness, some powders of rhubarb and magnesia mixed with an aromatic, and hence acquired a predilection for their use. The colour of this composition, doubtless, favoured the unfortunate mistake.

The family went to church on Sunday forenoon, leaving the convalescent to manage for himself. Having occasion, in the course of the day, to turn over the contents of a drawer, in which, during his sickness, the rhubarb powders had been deposited, he found a packet which he supposed to be of the same nature, and immediately determined on taking it. Thus, nearly two drachms of nitric oxide of quicksilver were unconsciously swallowed. The drug had been obtained by his father, several months previously, for the purpose of making a popular unguent; and, with culpable neglect, left in a situation accessible to all the younger branches of his family.

Extreme pain in the epigastric region, head-ache, constriction of the eye-lids, with dryness of the fauces and throat, were the immediate consequences of this deleterious dose. The poor young man was found, in the afternoon, stretched upon his bed, exhausted and almost speechless from pain and suffering. His parents could with difficulty comprehend his assurances that he knew nothing to which his present distress could be ascribed. By the opiate, however, a short remission from torture was obtained; and, during this, the inquisitive mother detected the true origin of the misfortune.

It was now my business to institute an appropriate plan of treatment with promptitude and circumspection. Repairing, therefore, to the residence of the young man with all possible expedition, I investigated attentively the phænomena and progress of the drug's baneful operation. He lay supine on the bed, speechless, and nearly insensible; his eyes were fixed, but the pupils still feebly contracted on the stimulus of light. The lower extremities were cold and clammy; the superior, dry and parched with febrile heat, occasionally subsultive. Respiration was hurried, fitful, and laborious; the mouth, tongue, and throat, rough and arid. The pulsations of the radial artery were thrilling, tense, innumerable; those of the tibial arteries were scarcely perceptible: the carotids beat 150 in a minute. All the regions of the abdomen were highly intolerant of pressure. The thirst was unquenchable. The superior parts of the trunk were bedewed with slight perspiration. General inquietude was painfully aggravated by the frequent recurrence of convulsive efforts to vomit. The visage exhibited a ghastly cadaverous appearance: the whole physiognomy strongly indicated the approach of death. From the beginning, the urinary excretion had been suspended. The fæcal matter, involuntarily evacuated, was, at this time, acrid, soapy, greenish-yellow-coloured, and disgustfully fœtid. No solid food had been taken, and even the deglutition of liquids was performed with difficulty.

The case appeared hopeless; yet I felt strongly inclined to make one last effort to save an interesting sufferer from destruction. Fourteen hours had now nearly elapsed from the introduction of the poison. The exhaustion of vital energy, consequent on its operation, was extreme. Hence, I felt somewhat embarrassed in arranging my remedial plan: it was adopted with caution and anxiety. All my exertions were amply repaid by its success. The irritable state of the stomach seeming to forbid the introduction of medicine by the mouth, an emollient injection, with castor oil, opium, and hyosciamus, was thrown up the rectum. The whole abdomen, meanwhile, was rubbed with the stronger liniment of ammonia; and the lower limbs, after being submitted to gentle friction, were wrapped in warm flannel, and placed in contact with bottles of hot water. In about half an hour the injection returned with a small quantity of discoloured fæces; and much flatus was discharged.

A dose of the decoction formerly mentioned, with the addition of sulphuric ether and compound tincture of castor, was now administered by the mouth. It excited distressful hiccough, which soon subsided; and the medicine was retained. After a short time, the same dose was repeated without producing uneasiness. Another injection, containing a double proportion of the opiate, was administered with good effect; and an increased dose of the tincture introduced into the stomach. At length, a calm succeeded the scene of agitation; and, during an hour, I watched the varying phænomena with increased solicitude.

Hitherto, no amelioration could be perceived in the circulating system. Although relieved from severe pain, my patient yet lay in a perilous condition. The present remission from suffering might be referred to diminished sensibility, consequent on the narcotic influence of the medicines administered, or to exhaustion originating from the continued operation of the malignant drug. His state had now become critical. Health or dissolution probably depended on the measures yet to be adopted.

With the intention of stimulating the cutaneous vessels, and of producing gentle perspiration, the abdomen was, a second time, rubbed with the ammoniated liniment; and flannel, imbued with the same, spread over the parts. Friction with it was also again employed to the inferior extremities; which were beginning gradually to recover their natural temperature. Port wine, diluted with warm water, was frequently administered in small quantities.

After remaining an hour and a half in the bowels, the injection returned nearly as the former had done. Soon afterwards, a genial moisture diffused itself over the trunk and lower limbs, and, by degrees, overspread his forehead. Respiration became more regular and tranquil. The pulsations of the radial artery were reduced to about one hundred and ten in a minute, and less tumultous, but hard and thrilling. The counting them, however, was rendered somewhat indistinct by the tremulous condition of the arm. The tongue had assumed the appearance which it displays in typhoid maladies. The torpor of the pupil was diminished; the physiognomical expression evidently improved. Less annoyance was denoted on examining the abdomen, except in the region of the bladder, which was tense and irritable.

My patient began, at this period, to feel a partial renovation of his physical energies. In a feeble tone, he solicited a little of some cooling beverage, and expressed his relief from the acute pain with which he had been tortured. Head-ache, however, with a vertiginous sensation, was now felt; and tension in the hypogastric region produced considerable distress. But hot fomentations were liberally applied to the part, and these soon succeeded in procuring a discharge of urine, at first scanty and painful; afterwards, free and copious, which added greatly to his comfort. The urine was high coloured, and turbid, affecting the urethra with a sense of prickly heat.

A third oily and anodyne injection was thrown up; the abdomen covered with the ammoniated cloth, and the tincture again administered. I remained half an hour after the exhibition of these: appearances were yet favourable. On my departure, I strongly enjoined perfect tranquillity, the liberal potation of toast and water or linseed tea; and requested that all the discharges might be reserved for inspection on my evening visit.

At seven, P. M. I found, with great pleasure, that appearances were still propitious. Soon after my departure, the young man had sunk into a disturbed slumber. He moaned heavily, and was agitated by frequent startings. The facial muscles, particularly those of the mouth, were slightly convulsed. Yet a mellow heat began by degrees to spread over the whole body, and was followed by profuse perspiration. The spasms now ceased, and the sleep became more restful and serene. After two hours, he awoke, apparently much refreshed. The injection was expelled, and was succeeded in an hour by considerable discharges of stool and urine. He had drunk freely, and felt much relieved, although exhausted and distressed by the vertiginous sensation on the slightest motion of the head. The fæcal matter, now evacuated, displayed nearly the same characters as the former; but was thinly intermixed with a mucous discharge. The external parts were much eroded by its acrimony. The urine was yet high coloured, deposited a lateritious sediment, and painfully affected the urethra on emission. The pulse was 100, softer, fuller, less agitated. The abdominal irritation was much tranquillized; but the patient pointed to the epigastrium as the seat of a hot distressful sensation, and still complained of thirst. The general diaphoresis continued.

Another glyster of starch, oil, and opium, was injected. Tepid milk, sweetened with sugar, and containing bread-crumb, was ordered to be sparingly but often taken; the former drink to be continued; wine to be moderately used, and a teaspoonful of the ether, with tincture of castor, to be administered about midnight. Friction of the abdomen and lower limbs was again prescribed, and the preservation of silence and tranquillity most rigidly enjoined.

The condition of my patient was little changed on Tuesday. He had slept several times during the night, but was not much refreshed by it, though more tranquil than on the preceding day. The injection had been expelled after a retention of three hours: the fæcal matter was improved both in colour and consistence; the urine was acrid, still high coloured; the pulsation of the radial artery ninety. Head-ache and abdominal irritation continued. The cutaneous surface was moist and mellow. Instead of the injections, castor oil was now prescribed, in small doses, twice or thrice a day. Continuation of the diluent drinks, wine, milk diet, and the abdominal friction, with an anodyne in the evening, was also directed.

It is useless to detail minutely the daily variations and treatment of this case. The same plan, occasionally modified, was continued. Cinchona was added to the wine: the drinks were acidulated with nitric acid, and the bowels regulated by castor oil, or the compound rhubarb pill. Eight days elapsed ere marked convalescence began; a month ere the patient walked abroad. At the end of three months from the unfortunate mistake, he resumed his former employment. At present, he is healthful and vigorous, and as capable of exertion as at any former period of his life.

May it not be supposed, that the debilitated state of the young man, at the time of his swallowing the deleterious oxide, signally favoured his escape from a situation thus perilous and desperate, by diminishing the predisposition to febrile excitement in the general system, and to inflammatory action in the gastric organ?*

* The nitric oxide of quicksilver, prepared by forming a saturated nitrate of that metal, and then decomposing the acid by heat, is an oxide of quicksilver, at the maximum of oxidation. It is more acrid than the red oxide prepared by the action of heat alone, and contains a small portion of nitric acid. Heated in a glass tube, it yields volatile metallic quicksilver; and oxigen gas is disengaged. By friction, it imparts to a clean copper plate a shining silvery hue. It is blackened by the action of hydro-sulphuret of ammonia, and converted into sulphuret of quicksilver. It is soluble in muriatic acid without heat, and forms the muriate of quicksilver (*deuchloride*). It may be distinguished from turbith mineral (*sul-sulphate of quicksilver*) by its not yielding sulphate of potass with a solution of the pure alkali. Very few cases of poisoning by the nitric oxide of quicksilver are on record. We never have observed one. It is, however, probable, that copious ingestion of warm diluent liquids by the mouth and rectum, general or local abstraction of blood, or both; intestinal evacuants, warm baths and fomentations, would, in the *earlier* stages of such a case, constitute a more successful plan of treatment, than uncertain and often dangerous attempts to decompose the poison in the stomach, by chemical re-agents.—EDITORS.

